# Fate of graft cells: what should be clarified for development of mesenchymal stem cell therapy for ischemic stroke?

**DOI:** 10.3389/fncel.2014.00322

**Published:** 2014-10-21

**Authors:** Yuka Ikegame, Kentaro Yamashita, Shigeru Nakashima, Yuichi Nomura, Shingo Yonezawa, Yoshitaka Asano, Jun Shinoda, Hideaki Hara, Toru Iwama

**Affiliations:** ^1^Department of Neurosurgery, Chubu Medical Center for Prolonged Traumatic Brain DysfunctionGifu, Japan; ^2^Department of Clinical Brain Sciences, Gifu University Graduate School of MedicineGifu, Japan; ^3^Department of Cell Signaling, Gifu University Graduate School of MedicineGifu, Japan; ^4^Department of Neurosurgery, Gifu University Graduate School of MedicineGifu, Japan; ^5^Department of Neurosurgery, Murakami Memorial Hospital, Asahi UniversityGifu, Japan; ^6^Department of Biofunctional Evaluation, Molecular Pharmacology, Gifu Pharmaceutical UniversityGifu, Japan

**Keywords:** mesenchymal stem cell, ischemic stroke, stem cell therapy, translational research, neurovascular unit

## Abstract

Mesenchymal stem cells (MSCs) are believed to be promising for cell administration therapy after ischemic stroke. Because of their advantageous characteristics, such as ability of differentiation into neurovascular lineages, avoidance of immunological problems, and abundance of graft cells in mesodermal tissues, studies regarding MSC therapy have increased recently. However, several controversies are yet to be resolved before a worldwide consensus regarding a standard protocol is obtained. In particular, the neuroprotective effects, the rate of cell migration to the lesion, and differentiation direction differ depending on preclinical observations. Analyses of these differences and application of recent developments in stem cell biology or engineering in imaging modality may contribute to identification of criteria for optimal stem cell therapy in which reliable protocols, which control cell quality and include safe administration procedures, are defined for each recovery phase after cerebral ischemia. In this mini review, we examine controversies regarding the fate of grafts and the prospects for advanced therapy that could be obtained through recent developments in stem cell research as direct conversion to neural cells.

## DEVELOPMENT OF MESENCHYMAL STEM CELL THERAPY STUDY FOR ISCHEMIC STROKE

Ischemic stroke is a common central nervous system (CNS) disease. Despite continuous development in treatments, stroke is still a major cause of death or disability, and therefore, more effective therapies are required. In 1990s, clinical trials neuroprotective agents targeted single mechanism, i.e., glutamate-induced neurotoxicity revealed to become failure (Hoyte et al., 2004). In the lesion insulted by brain ischemia, multiple pathogenic mechanisms are activated. As the failures in the early neuroprotective drug development showed (Degraba and Pettigrew, 2000), a genuine effective therapy would be required to solve the pleiotropic pathology (Teng et al., 2008; Guo and Lo, 2009).

Another concept to treat lost function by ischemia is to supply cells or tissue for replacement of the damaged brain tissue. In the early days of stem cell research, stem cells were expected as a source of tissue regeneration. Since the publication of the earliest reports of attempted administration of embryonic or neonatal neural stem cells for regeneration of the CNS in the early 1990s (Renfranz et al., 1991; Snyder et al., 1992), diverse cell types have been investigated to identify an ideal cell line to generate tissue grafts for CNS. Candidate cells can be categorized into embryonic, fetal, neonatal, or adult by maturation of each origin tissue. When categorized by a stage of differentiation, the examined cells can be sourced from pluripotent cells (embryonic stem cells or induced pluripotent cells), ectodermal lineage (neural stem cells, olfactory neuroepithelial stem cells, or NT2 cell line derived from neuroteratocarcinoma), mesodermal lineage [mesenchymal stem cells (MSCs), CD34+ cells, endothelial progenitor cells, hematopoietic stem cells, or bone marrow mononuclear/stromal cells]. As discussed in published reviews on stem cell therapies (Locatelli et al., 2009; Bhasin et al., 2013; Kalladka and Muir, 2014), neural stem cells, and mesodermal lineage listed above have already been applied for ischemic stroke in clinical settings from subacute phase to chronic phase.

In this mini review, the advantages of MSCs, as a source for stem cell therapy, are summarized. Furthermore, controversial points in preclinical experimental studies and the developing field of MSC therapy resulting from the recent evolution in stem cell biology are discussed by focusing on the biological features of mesenchymal stem cells (MSCs).

Among stem cell therapies, the greatest numbers of clinical trial for MSC have been conducted (Rosado-De-Castro et al., 2013a), thus MSC therapy can be the most practical stroke treatments in cell-based therapies (Eckert et al., 2013). More than 30 years after when Friedenstein et al. (1966) isolated osteogenic cell population from bone marrow, MSCs have been identified in bone marrow (Pittenger et al., 1999), adipose tissue (Zuk et al., 2002), umbilical cord (Erices et al., 2000), peripheral blood (Ukai et al., 2007), dental pulp (Gronthos et al., 2000), and a wide range of mesodermal tissues including perivascular site in brain (Kang et al., 2010; Paul et al., 2012). The criteria for identifying MSCs as proposed by the Mesenchymal and Tissue Stem Cell Committee of the International Society for Cellular Therapy are (1) plastic adherence of isolated cells in culture; (2) in cell surface marker analysis, >95% of the culture positively expressing the cell surface markers CD105, CD73, and CD90, while being negative for CD34, CD45, CD14, or CD11b, CD79a, or CD19, and human leukocyte antigen-DR; and (3) *in vitro* differentiation into three mesodermal cell types, namely osteoblasts, adipocytes, and chondroblasts (Dominici et al., 2006). Moreover, the characteristics of MSC present advantages. MSC have been shown their multipotency that is beneficial to differentiate into multiple lineages to repair neurovascular unit or neural network; they could demonstrate multiphasic actions to modify endogenous repairing process including reprogramming, harmful immune response, or chemical reactions via secretion abilities; they are easier to prepare for grafting due to their accessible cell source and proliferation potential for rapid cell expansion. (Doeppner and Hermann, 2010; Grande et al., 2013; Wan et al., 2013)

The first series of successful experiments for MSCs for the treatment of ischemic stroke was reported by Chopp’s group (Chen et al., 2000; Li et al., 2000; Zhang et al., 2000). They have examined multiple protocols for bone marrow stromal-derived stem cells (BMSCs) such as administration route (intracerebral, transventricular, intra-arterial, transvenous), timing, or dose, as well as have analyzed mechanisms of functional recovery focused on restore or remodeling functional connectivity in neural circuits/tract. Subsequently, details required for the establishment of safe and effective therapy protocols (Borlongan, 2009; The STEPS Participants, 2009; Savitz et al., 2011) have been analyzed by a number of investigators. Most results in the preclinical studies have indicated that MSC administration is beneficial. In this context, clinical trials employing systemic administration via peripheral veins were initiated more recently (Lee et al., 2010; Honmou et al., 2011). So far, these trials have not demonstrated severe adverse results (Lalu et al., 2012), even during observation periods lasting longer than a few years, despite the prediction of risks, such as embolization (Ge et al., 2014; Yavagal et al., 2014), infection, and tumorigenesis (Coussens et al., 2000; Li et al., 2007), in experimental studies.

## CONTROVERSIES IN PRECLINICAL STAGE

Overall, accumulated findings have indicated that MSC therapy is reliable for stroke treatment. However, several points must be clarified for achievement of consensus as a reliable protocol. As shown in **Table 1**, the conditions of some preclinical studies resulted in differing outcomes because of graft cell detection in the lesion, infarct volume reduction, functional recovery, marker expression (neuronal, glial, or vascular: direction of differentiation), and the type of MSCs considered to have more therapeutic effects, particularly BMSCs and adipose tissue-derived stem cells (ASCs).

**Table 1:** Examles of precilinical reports present discrepancy in results.

	Graft cell detection in brain		Infarct volume reduction		Functional recovery		Differentiation in the lesion		
Administration route	Yes	No	Yes	No	Yes	No	Neuronal	Oligodendro-glial	Vascular
Intra-arterial	Shen et al. (2006; BM, A)		Ishizaka et al. (2013; BM, A)	Jiang et al. (2014; AT, S)	Shen et al. (2006; BM, A)		Jiang et al. (2014; AT, S)	Shen et al. (2006; BM, A)	
	Ishizaka et al. (2013; BM, A) Jiang et al. (2014; AT, S)				Jiang et al. (2014; AT, S)			Jiang et al. (2014; AT, S)	
Intravenous	Chen et al. (2001; BM, A)	Ikegame et al. (2011; BM, AT, A)	Leu et al. (2010; AT, A)	Steiner et al. (2012; BM, A)	Chen et al. (2001; BM, A)	Steiner et al. (2012; BM, A)	Chen et al. (2001; BM, A)	Li et al. (2005; BM, S)	Leu et al. (2010; AT, A)
	Li et al. (2005; BM, S)		Ikegame et al. (2011; BM, AT, A)	Gutierrez-Fernandez et al. (2013; BM, AT, A)	Li et al. (2005; BM, S)		Wei et al. (2012; BM, A)		Wei et al. (2012; BM, A)
	Leu et al. (2010; AT, A)	Gutierrez-Fernandez et al. (2013; BM, AT, A)	Honmou et al. (2012; BM, A, S, C)		Leu et al. (2010; AT, A)		Honmou et al. (2012; BM, A, S, C)		
	Wei et al. (2012; BM, A)				Ikegame et al. (2011; BM, AT, A)				
	Honmou et al. (2012; BM, A, S, C)				Wei et al. (2012; BM, A)				
	Steiner et al. (2012; BM, A)				Gutierrez-Fernandez et al. (2013; BM, AT, A); Honmou et al. (2012; BM, A, S, C)				
Intracerebral	Chen et al. (2000; BM, A) Kubis et al. (2007; AT, A)				Chen et al. (2000; BM, A)		Chen et al. (2000; BM, A)		Kubis et al. (2007; AT, A)

*BM, bone marrow-derived MSCs; AT, adipose tissue-derived MSCs; A, acute phase infusion; S, subasute phase infusion; C, choronic phase infusion.*

### MIGRATION TO THE LESION

A major discrepancy in the results of preclinical studies is whether graft cells have the ability to migrate to a cerebral lesion, although mechanisms of MSC transmigration across the blood–brain barrier (BBB) have been analyzed (Liu et al., 2013). The accumulation of graft cells in the lesion is expected to directly enhance neuroprotection and cell replacement in infarcted tissue. A comparison of different administration routes revealed that transarterial delivery was more successful in order to detect graft cells in the brain than transvenous delivery, although several studies reported a decrease in the number of detected cells in the later phase (Ishizaka et al., 2013; Mitkari et al., 2013). The transvenous route induced fewer side effects than intra-arterial infusion; however, physiologically, graft cells must pass through several traps, such as the lung and BBB. Although, the BBB can be disrupted by ischemic insult around the damaged areas, MSCs may have the basic ability to transmigrate the BBB as immune cells in response to homing signals to the lesion (Liu et al., 2013). Nonetheless, there are certainly successful examples demonstrating the integration of graft cells in the peri-infarct area even after transvenous infusion from a peripheral vessel (**Table 1**).

Classically, immunohistological analysis is a standard method to detect MSC migration, but recent imaging techniques, such as magnetic resonance imaging (MRI) with magnetic cell labeling (Detante et al., 2012; Canazza et al., 2013) and nuclear imaging using ^99m^Tc-labeled graft (Detante et al., 2009; Vasconcelos-Dos-Santos et al., 2012), have been proposed to reveal the distribution of MSCs. Subsequently, a phase I clinical trial employing ^99m^Tc – single photon emission computed tomography (SPECT) for assessment of biodistribution of the labeled grafts in subacute patients have safely conducted (Rosado-De-Castro et al., 2013b). The findings of these recent analytical methods may resolve the question of accurate distribution of graft cells.

### FUNCTIONAL RECOVERY

Many preclinical studies have also reported differences in infarct volume reduction and functional recovery (Hao et al., 2014). Assessment methods of functional recovery vary, although there certainly are popular tests in animal studies, such as the treadmill test or Roger’s test. Therefore, differences in functional assessment may simply be based on differences in the employed assessment methods. On the other hand, it is more difficult to elucidate discrepancies in infarct volume reduction. *In vivo* studies with rodents have been conducted to investigate the changes in infarct volume reduction by direct measurement of the brain tissue after decapitation. Regarding clinical applications, non-invasive methods, such as MRI, may be beneficial to translate the findings of *in vivo* studies to clinical settings. Although the availability of mechanical devices varies among laboratories, the development of alternative clinical methods is recommended for future *in vivo* experiments.

Another problem is whether MSCs isolated from different tissues also differ. MSCs are obtained from diverse mesodermal tissues, i.e., bone marrow, adipose tissue, dental pulp, or cord blood. MSCs from different sources show different characteristics *in vitro* (Kern et al., 2006; Hsiao et al., 2012). Therefore, comparative study for different cell sources as conducted by Gutierrez-Fernandez’s group is important, however, the therapeutic effects in similar experimental ischemic stroke models also differ in transvenous administration studies (Ikegame et al., 2011; Steiner et al., 2012; Gutierrez-Fernandez et al., 2013) compared to intra-arterial administration studies that have shown graft cells in the lesion. (**Table 1**)

On the other hand, nuclear imaging is another available method to assess the therapeutic effectiveness. Diffusion and perfusion-weighted imaging provide information of blood supply in the brain (Canazza et al., 2013). Furthermore, functional MRI is employed by experimental studies in rodents, which unable to assess functional recovery (Suzuki et al., 2013) and even neural network by analyses of resting state functional MRI (Canazza et al., 2013). The neural integrity has been investigated by ^123^I – Iomazenil SPECT (Saito et al., 2013). A ^18^F-FDG positron emission tomography study have measured glucose metabolism after MSC therapy in rats for cerebral ischemia (Miyamoto et al., 2013). For assessment of functional recovery, these methods from more bio-functional aspect would be practical in addition to observations of behavioral change.

### DIRECTION OF DIFFERENTIATION

The direction of differentiation also remains controversial for *in vivo* experimental studies. Although MSCs are derived from mesenchymal tissue, they exhibit multipotency and transdifferentiation into ectodermal lineages, including neural cells, both *in vitro* and *in vivo* (Zuk, 2013). Previous *in vitro* immunochemistry studies have demonstrated the ability of MSCs to differentiate into cell types that comprise the neurovascular unit, including neurons, astrocytes (Wislet-Gendebien et al., 2004), and endothelial cells (Hess et al., 2002; Planat-Benard et al., 2004). Moreover, possible differentiation abilities toward oligodendrocyte lineage (NG2-positive cells; Shen et al., 2006), specific types of neurons, such as glutamatergic neurons (Yu et al., 2014), and smooth muscle cells of vessels (Kubis et al., 2007) have been demonstrated. *In vivo* studies have reported that graft cells detected in the lesion result from neuronal or glial differentiation (Guzman et al., 2008). However, one study demonstrated the vascular fate rather than differentiation to neural lineages (Kubis et al., 2007).

To ensure the practical differentiation, in addition to these morphological, immunohistochemical, or genetic assessments, cells should be further examined. With respect to neural differentiation, neurotransmitter responsiveness or electrophysiological recording is required to examine their function as a neuron (Yang et al., 2011). Moreover, when MSCs are employed, absence of cell fusion also should be excluded. Though the MSC’s rate of spontaneous cell fusion is only 2–11 clones per million cells (Terada et al., 2002), and the mechanism may also participate in the tissue repair, nonetheless, biologically it should be distinguished from differentiation. BMSC and ASC are observed the neural differentiation that can show neural function in earlier studies. First, Ashjian et al. (2003) recorded K^+^ current on neuronal cells induced from ASC. Cho et al. (2005) reported synaptic transmission, and Wislet-Gendebien et al. (2005) showed action potential of the neuron-like cells differentiated from BMSC.

### AUTOLOGOUS OR ALLOGENIC?

With the exception of the acute phase after ischemic insult, both allogenic and autologous grafting of MSCs can be prepared. Although the efficacy of technologies has improved, besides the advantage of MSCs in immunomodulation, theoretically allogenic grafts cannot ameliorate all concerns regarding transinfection or immunological side effects. Autologous grafts can overcome the problems related to allogenic cells. Nonetheless, at the present stage, other than obtaining the major MSCs, the use of both BMSCs and ASCs requires invasive procedures. Bone marrow aspiration and harvesting of adipose tissue are considered safe and established techniques; however, because ischemic stroke patients usually take antiplatelet or anticoagulant agents, and in some case, the patient may be intolerant to other conditions, further less invasive methods, such as the use of peripheral blood, present alternative sources of cells. As mentioned in the previous section, each type of MSCs from different cell sources tend to exhibit original traits or abilities, although they meet the criteria of MSCs. Knowledge regarding defined factors/conditions for MSC-fate regulation could enable the preparation of homogenous MSCs, even from peripheral blood (Meng et al., 2013).

Autologous grafts may have an additional advantage over allogenic grafts. In preclinical observations, MSCs reportedly developed function following contact with a conditioned media (Egashira et al., 2013), serum (Honmou et al., 2011), or cerebrospinal fluid from patients (Orito et al., 2010), which is reflected in the biological responses to invasive stimulation. It is possible that MSCs may achieve proper function in reaction to insults (Kurozumi et al., 2005; Xin et al., 2013). Therefore, graft cells harvested from ischemic stroke patients may gain more favorable function than allogenic grafts from those who are not affected by ischemic insults. Strikingly, the first nonrandomized clinical trial for a protocol with autologous BMSCs and serum has been shown to be safe and effective (Bang et al., 2005; Lee et al., 2010; Honmou et al., 2011). A 5-year randomized trial also began in 2012, which will provide further information regarding autologous stem cell therapy (Kim et al., 2013).

## POSSIBILITY OF ADVANCED MSC THERAPIES AS A SOLUTION OF QUESTIONS

### MSC MODIFICATION AND IDENTIFICATION BY DEFINED FACTORS RELATED TO CELL FATE REGULATION

From a pharmacological viewpoint, the actions of agents should be confirmed after administration. If MSCs are regarded as a type of biological drug, then differences in differentiation ability should be better clarified.

Emerging induced pluripotent stem cells (iPSC) studies have shown promising benefits in the field of regenerative medicine that could have at least two major impacts on MSC studies. These findings may be useful to settle the controversies listed above, particularly those regarding the direction of differentiation of graft cells in the host and differences in the characteristics of MSCs originating from the cell source.

First, the appearance of iPSCs indicates the potential of multipotency in somatic cells (Takahashi and Yamanaka, 2006), which is supported by observations of differentiation into either neural or endothelial cells in MSCs. Although many reports have demonstrated the ability MSCs of mesodermal origin to differentiate into other type of germ cells of ectodermal lineages (neural cells) and endodermal lineages (insulin-producing cells), which could indicate multipotency, the defined conditions for MSCs to differentiate into neural cells remain uncertain. In the infancy of stem cell research, cell fusion and contamination of neural crest cells were suggested as the mechanism of a graft cell to express neural markers in the host tissue after cell administration (Wrage et al., 2008; Maltman et al., 2011). If the postulates reveal to be the main mechanism, neural marker expression can’t be called neural differentiation, which unable MSC to be called “stem cell.” Therefore, until recently, the term “MSC” containing the term “stem cell” had its pros and cons, and thus, MSCs were called stromal cells. However, successful reprogramming of skin fibroblasts to the multipotent state has provided more information to support the multipotency of MSCs.

Second, induction techniques may contribute to further elucidate the quality control mechanisms for the use of MSCs. Protocols for chemical induction to neuron or glia had been developed recently (Safford and Rice, 2005; Franco Lambert et al., 2009; Yu et al., 2011). Following the publication of methods to harness and propagate iPSCs, other methods related to direct conversion from fibroblasts to neuronal cells by defined transcription factors have been reported (Vierbuchen et al., 2010; Yang et al., 2013). The neural lineage is composed of induced neuronal (iN) cells, induced neural progenitor cells (iNPCs), and induced NSCs (iNSCs; Yang et al., 2011; Abdullah et al., 2012; Corti et al., 2012; Shi and Jiao, 2012). Moreover, iPSC-derived MSCs (iPSC–MSCs) were identified (Jung et al., 2012). There are multiple pathways for neural induction. As listed in the **Table 2**, in addition to defined transcriptional factors for direct conversion, microRNA (Feng and Feng, 2011; Pham and Gallicano, 2012; Bian et al., 2013) or other epigenetic factors (Namihira and Nakashima, 2011) can contribute to differentiation. The definitive conditions to propagate/identify iN cells, iNSCs, iNPCs, or iPSC–MSCs may be useful to propose a standard protocol for the required type of MSCs.

**Table 2:** Suggested factors related to cell fate regulation for direct conversion.

	Transcriptional factors	microRNA
	*(Yang et al., 2011; Abdullah et al., 2012; Kim et al., 2012; Lujan and Wernig, 2012; Shi and Jiao, 2012)	*(Feng and Feng, 2011; Pham and Gallicano, 2012; Bian et al., 2013)
**NSC**	Brn4/Sox2/Klf4/c-Myc/E47/Tcf3; Brn2/Ascl1/Myt1L; Sox2; Oct4/Sox2/Klf4/c-Myc	miR-134, miR-195, miR-184, miR-125, miR-137
**NPC**	Sox2/Oct4/Klf4/c-Myc; Brn2/Ascl1/Myt1L; Ascl1/Ngn2/Hes1/Id1/Pax6/Brn2/Sox2/Klf4/c-Myc; FoxG1/Sox2; Brn2/FoxG1/Sox2; Brn4/Sox2/Klf4/c-Myc	
**Neuron**	Brn2/Ascl1/Myt1L/miR-124; Brn2/Ascl1/Myt1L/NeuroD1; Ascl1/Myt1L/NeuroD2/miR-9/9*/miR-124; Brn2/Ascl1/Ngn2; Brn2/Ascl1/Myt1L/NeuroD1/Zic1	Let-7b, miR-125b, miR-9, miR-137, miR-124, miR-17, miR-92, miR-106
Dopaminergic neurons	Ascl1/Lmx1a/Nurr1; Ascl1/Lmx1a/Nurr1/Brn2/Mytl1L/FoxA2	miR-133b, miR-132, miR-7a
Spinal motor neurons	Brn2/Ascl1/Myt1L/NeuroD1/Lhx3/Hb9/Isl1/Ngn2	miR-17-3p, miR-9
**Glia**		
Astrocyte	FoxG1/Sox2; Sox2/Oct4/Klf4/c-Myc	miR-125b, miR-24, miR-29
Oligodendeoglial precursur cell	Sox2/Olg2/Zfp536 (Yang et al., 2013)	miR-7, miR-219, miR-23, miR-338

*Review articles.

### ORGANOGENESIS FOR TISSUE REPLACEMENT

Lancaster et al.’s (2013) team developed a three-dimensional brain tissue from iPSCs by the floating culture method. To obtain functional recovery *in vivo*, several groups have shown that tissue regeneration or replacement of damaged tissue with *ex vivo* materials is not always necessary (**Table 1**). Particularly in the brain tissue, repair of the neural circuitry is required to improve function. Nonetheless, tissue engineering using scaffolds (Mahmood et al., 2013) or novel organogenesis methods present possible transplantation treatments to recover neurological deficits.

## CONCLUSION

Since the first report of MSC (Pittenger et al., 1999), investigators have revealed favorable cell characteristics for cell therapies and have shown evidence for feasible stem cell therapy using MSCs in order to achieve safe applications in clinical settings. However, there are limited methods to ensure reliable treatment. Nevertheless, further studies combined with developments in other biological and/or engineering fields may solve these present problems, and establish an ideal stem cell therapy beyond categorization of MSCs.

## Conflict of Interest Statement

The authors declare that the research was conducted in the absence of any commercial or financial relationships that could be construed as a potential conflict of interest.
